# The involvement of FOXO1 in cytotoxic stress and drug-resistance induced by paclitaxel in ovarian cancers

**DOI:** 10.1038/sj.bjc.6604279

**Published:** 2008-03-04

**Authors:** T Goto, M Takano, J Hirata, H Tsuda

**Affiliations:** 1 Department of Gynaecologic Oncology, Saitama Medical University International Medical Centre Comprehensive Cancer Centre, 1397-1 Yamane, Hidaka city, Saitama 350-1298, Japan; 2 Department of Obstetrics and Gynaecology, National Defense Medical College, 3-2 Namiki, Tokorozawa, Saitama 359-8513, Japan; 3 Department of Pathology II, National Defense Medical College, 3-2 Namiki, Tokorozawa, Saitama 359-8513, Japan

**Keywords:** ovarian cancer, paclitaxel, FOXO1, drug resistance, reactive oxygen spices (ROS)

## Abstract

The role of transcriptional factor FOXO1 in the mechanism of drug-resistance in ovarian cancer has not been elucidated. In ovarian cancer cell lines, FOXO1 expression and its correlation with paclitaxel treatment was investigated by cytotoxic assay and silencing experiment. Clinical ovarian cancer samples were also examined for FOXO1 expression by immunohistochemistry. FOXO1 expression was distinctively upregulated in paclitaxel-resistant cell line, and enhanced by exposure to paclitaxel. FOXO1 overexpression was frequently observed in tissue samples from chemoresistant patients compared to chemosensitive patients. FOXO1 silencing in paclitaxel-resistant cell line decreased its resistance. Modification of oxidative stress by co-treatment with pharmacologic modulators of reactive oxygen species attenuated cytotoxicity of paclitaxel. Downstream targets of FOXO1 involving oxidative stress were also attenuated in silencing experiment, suggesting its involvement in altered sensitivity to paclitaxel. These results indicate that FOXO1 links to cytotoxic stress induced by paclitaxel and contributes to the drug-resistance in ovarian cancers.

Paclitaxel is one of the most active cancer chemotherapeutic agents known. It is effective against a variety of human tumours, including ovarian carcinomas (Rowinsky and Donehower, 1995; McGuire *et al*, 1996; Wiseman and Spencer, 1998). The efficacy of paclitaxel is limited by intrinsic or acquired drug resistance in a population of surviving malignant cells. One molecular mechanisms for acquired tumour cell resistance to paclitaxel is explained by overexpression of the drug efflux pump MDR-1; however, the role of this is still undetermined.

The mammalian FOXO family of Forkhead transcription factors, consisting of FOXO1, FOXO3a, and FOXO4, is a direct downstream target of the PI3K/Akt pathway (Brunet *et al*, 1999; Kops *et al*, 1999). Post-translational modification of FOXO proteins is an important mechanism that regulates the ability of different transcription factors to activate distinct gene sets, involved in cell cycle inhibition (Dijkers *et al*, 2000), apoptosis (Sunters *et al*, 2003), defense against oxidative stress and DNA repair (Kops *et al*, 2002; Nemoto *et al*, 2004). Since FOXO proteins were reported to be critical mediators of apoptosis in cytotoxicity inducing drugs in many cells (Sunters *et al*, 2003; Kajihara *et al*, 2006; Goto *et al*, 2008), we postulated that FOXO expression or transcriptional activity could be important event in the drug sensitivity in cancer cells. In the present study, we examined the consequence of FOXO1 expression correlating with paclitaxel cytotoxicity and sensitivity in ovarian cancer cell lines using parent cells and paclitaxel-resistant derivative cells, and confirmed its expression in clinical samples from chemosensitive and resistant patients. Furthermore, we explored the possible underlying mechanism in involvement of FOXO1 with paclitaxel resistance.

## MATERIALS AND METHODS

### Cell lines, culture conditions and treatment

KF28 is a single-cell clone of the human ovarian carcinoma cell line KF. KFr13 is a cisplatin-resistant subline derived from KF28 cells as described previously (Kikuchi *et al*, 1986), and KFr13Tx is a paclitaxel-resistant subline derived from KFr13 cells (Yamamoto *et al*, 2000a). These cell lines were grown as monolayer cultures in RPMI-1640 (Immuno-Biological Laboratories Co. Ltd, Gunma, Japan) medium supplemented with 10% fetal bovine serum (Invitrogen Japan KK, Tokyo, Japan), 2 mM glutamine, 100 U penicillin per ml, and 100 *μ*g streptomycin per ml (Invitrogen Japan KK) in a humidified atmosphere of 5% CO_2_ at 37°C, and routinely tested for mycoplasma infection. Paclitaxel was obtained from Bristol Meier's Squib Oncology (Tokyo, Japan) and dissolved in dimethylsulphoxide .

### Cell proliferation and cytotoxicity assay

Ovarian cancer cells were seeded onto 96-well plates, at approximately 2 × 10^3^ or 10 × 10^3^ cells cm^−2^ for proliferation or cytotoxicity assays, respectively, and allowed to attach overnight. Cell viability was determined by MTS assay using the CellTiter 96 aqueous one solution cell proliferation assay (Promega KK Japan, Tokyo, Japan) according to the manufacturer’s instructions. To study the effects of paclitaxel on cell proliferation, cells were treated with various doses of paclitaxel for 24 h. After completion of the treatment, the percentage absorbance was calculated against untreated cells. For growth curve analysis and Trypan blue exclusion test, ovarian cancer cells were plated in 24-well plates (2 × 10^3^ or 10 × 10^3^ cells cm^−2^). At the indicated time points, cells were trypsinised to detach from the plates and stained with Trypan blue (Doujin, Kumamoto, Japan), and cell number was counted under a microscopy using a haemocytometer. Each experiment was performed in quadruplicate. Flow cytometry analysis was used to quantify apoptosis in ovarian cancer cells by evaluating the sub-G1 fraction (<2 N) after propidium iodide (PI) staining of ethanol-fixed cells.

### Western blotting analysis

Protein concentrations were determined by Bradford assay (Bio-Rad Laboratories, Hercules, CA, USA) and equal amounts of whole cell extracts or nuclear and cytoplasmic protein fractions were separated on a 10% SDS–polyacrylamide gel before electrotransfer onto a polyvinylidene diflouride membrane (Hybond P, GE Healthcare UK Ltd, England, UK). Nonspecific binding sites were blocked by overnight incubation with 5% dried skimmed milk in Tris-buffered saline (130 mM NaCl, 20 mM Tris, pH 7.6). Primary antibodies to FOXO1, phospho-FOXO1 (Ser256), Akt, phospho-Akt (Ser473) (Cell Signaling Technology, Boston, MA, USA), FOXO3a (Upstate, Temecula, CA, USA), FOXO4, GADD45*α*, MnSOD, catalase, p27^Kip1^, Lamin B1 (Santa Cruz Biotechnology Inc., Santa Cruz, CA, USA), and PARP cleavage site (214/215) (Biosource, Carmavillo, CA, USA) were used at 1 : 1000 whereas the antibody to *β*-actin (Abcam, Cambridge, UK) was diluted 1 : 100 000. Primary antibodies were detected using horseradish peroxidase linked anti-mouse, anti-goat or anti-rabbit conjugates as appropriate (Dako Cytomation, Kyoto, Japan), and visualised using the ECL detection system (GE Healthcare UK Ltd, England, UK).

### Real-time quantitative–PCR

Total RNA was extracted from ovarian cancer cell lines by a ready-to-use reagent (TRIZOL, Invitrogen Japan KK) according to the manufacturer’s instructions and reverse transcribed using the Superscript III reverse transcriptase (Invitrogen Japan KK), and resulting first-strand cDNA was used as template in the real-time quantitative–PCR (RTQ–PCR) analysis. The following gene-specific primer pairs were used: L19-sense (5′-GCGGAAGGGTACAGCCAAT-3′) and L19-antisense (5′-GCAGCCGGCGCAAA-3′); FOXO1-sense (5′-TGGACATGCTCAGCAGACATC-3′) and FOXO1-antisense (5′-TTGGGTCAGGCGGTTCA-3′). L19, a non-regulated ribosomal housekeeping gene, served as an internal control and was used to normalise for differences in input RNA. Detection of the transcripts was performed with Power SYBR Green (Applied Biosystems, Foster City, CA, USA) and an ABI PRISM 7700 sequence detection system according to the manufacturer's recommended protocol (Applied Biosystems). All measurements were performed in triplicate.

### Patient selection for immunohistochemical staining

Of patients with primary epithelial ovarian cancer treated at the National Defense Medical College Hospital (Saitama, Japan), the following patients were selected: (a) patients who received no chemotherapy before any surgical therapy; (b) patients who harboured measurable residual tumours after initial debulking surgery; (c) patients who were treated with six courses of adjuvant chemotherapy using paclitaxel (180 mg m^−2^) and carboplatin (AUC=5) chemotherapy after the initial surgery and (d) patients who agreed to participate in the current study with written informed consent. The patients were divided into the following four groups according to their response to chemotherapy measured with CT or MRI: (a) CR (complete response) group; (b) PR (partial response) group; (c) SD (stable disease) group and (d) PD (progressive disease) group. Responders were defined as patients with CR or PR, and non-responders were defined as those with SD and PD. A total of 13 responders and 10 non-responders were included in the study.

### Immunohistochemistry

After reviewing the haematoxylin-stained sections, a paraffin block of the most representative sections were selected and cut into a 4-*μ*m thickness. All of the sections were deparaffinised and rehydrated with xylene and a graded alcohol series. To inactivate endogenous peroxidase activity, sections were immersed in methanol containing 0.3% hydrogen peroxide for 30 min at room temperature, then incubated in 2.0% blocking serum for the reduction of nonspecific binding. The sections were incubated with primary antibodies against FOXO1 (1 : 50 dilution; Cell Signaling Technology, Boston, MA, USA) and MnSOD (1 : 50 dilution; Santa Cruz Biotechnology Inc.) in humid chamber for 60 min at room temperature, followed by washing with PBS. For the visualisation of FOXO1 and MnSOD, the EnVision^+^™ system (Dako Cytomation) was applied to the sections for 2 h at room temperature, and diaminobenzidine hydrochloride was used. These sections were counterstained with Meyer’s haematoxylin. Cytoplasmic staining was considered as positive expression. The proportion of positive-stained cells was counted in more than 10 high power fields by two investigators who were blinded to the data of patient characteristics. Immunostaining for the specimen was classified as positive when >10% of cells were positive.

### Transient transfection

For silencing experiments, KFr13Tx cells cultured in six-well plates were transfected with 50 nM of FOXO1 si*GENOME* SMARTpool or non-targeting siRNA pool (Dharmacon, Lafayette, CO, USA) using Lipofectamine 2000 (Invitrogen Japan KK) according to the manufacturer’s specifications. FOXO1 knockdown was confirmed by western blot analysis in all the experiments.

### Intracellular reactive oxygen species measurement

Levels of intracellular H_2_O_2_ were assessed spectrofluorimetrically using 5-(and-6)-carboxy-2′,7′-dichlorodihydrofluorescein diacetate (carboxy-H_2_DCFDA, Invitrogen Japan KK) according to the manufacturer’s instructions. Briefly, cells were seeded and attached overnight on 96-well plates (2 × 10^4^ cells cm^−2^) and were washed with PBS and initially incubated with 10 *μ*M carboxy-H_2_DCFDA in PBS for 30 min, then changed to paclitaxel or H_2_O_2_ at the indicated concentrations with carboxy-H_2_DCFDA in PBS concomitantly. After 4 h incubation, cells were washed with PBS and fluorescence intensity was measured by spectrofluorometry. Excitation and emission wavelengths used were 485 and 525 nm, respectively. The relative H_2_O_2_ production induced by paclitaxel or H_2_O_2_ was expressed as the ratio between fluorescence intensity in cells treated with paclitaxel or H_2_O_2_ and with PBS alone.

### Statistical analysis

All values are presented as mean±s.d. Statistical significance between two groups was determined by use of a two-tailed *t*-test and values of *P*<0.05 were considered significant.

## RESULTS

### Cellular characterisation of ovarian cancer cell lines

**Figure 1:**
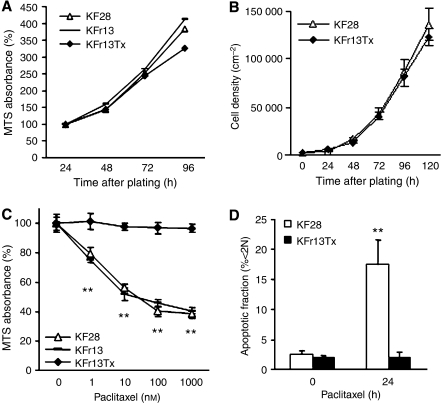
Cellular characterisation of ovarian cancer cell lines. (**A**) KF28, KFr13 and KFr13Tx cells were seeded in 96-well plates and the relative increase in cell viability was monitored by MTS assay at the indicated time points. (**B**) KF28 and KFr13Tx cells were plated in 24-well plates for growth curve analysis at the indicated time points. Each experiment was performed in quadruplicate. (**C**) KF28, KFr13 and KFr13Tx were incubated with the indicated concentrations of paclitaxel and cell viability was determined by MTS assay 24 h later. (**D**) KF28 and KFr13Tx cells treated with 10 nM paclitaxel were harvested at the indicated time points and analysed by flow cytometry to determine the apoptotic cell fractions. The results show mean±s.d. of triplicate measurements and ^**^P<0.001.

To examine the role of FOXO transcriptional factor in ovarian cancer cells, cellular characteristics, such as proliferation ability and drug sensitivity, were first confirmed in three representative ovarian cancer cell lines, parent cells KF28, cisplatin-resistant derivative and paclitaxel-resistant derivative cells, KFr13 and KFr13Tx. Cellular proliferation abilities in three cell lines were comparable as determined by MTS assay (Figure 1A), which was confirmed by growth curve analysis for KF28 and KFr13Tx (Figure 1B). Drug sensitivity to paclitaxel was re-examined by MTS assay after 24 h exposure, which revealed considerable acquired resistance only in KFr13Tx cells (Figure 1C). These findings were also confirmed for KF28 and KFr13Tx cells treated with 10 nM paclitaxel for 24 h by flow cytometry of PI-stained cells (Figure 1D).

### Differential expression of FOXO1 in ovarian cancer cell lines

**Figure 2:**
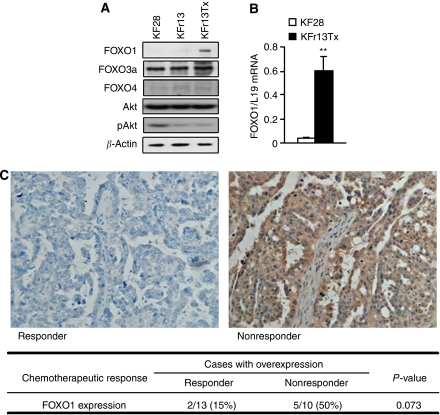
Differential expression of FOXO1 in drug sensitive and resistant ovarian cancer cell lines and ovarian cancer samples. (**A**) Comparative analysis of FOXO1, FOXO3a, FOXO4, total and phosphorylated Akt (pAkt) expression in KF28, KFr13 and KFr13Tx cells by western blot analysis. *β*-Actin served as a loading control. (**B**) RTQ–PCR analysis demonstrated significant higher FOXO1 mRNA levels in KFr13Tx cells when compared to KF28 cells. The results show mean±s.d. of triplicate measurements and ^**^P<0.001. (**C**) The representative immunohistological staining of FOXO1 of a chemotherapy-responder and non-responder is shown. The adjacent table summarises the number of patients with immunohistochemical reactivity of FOXO1 according to the chemotherapeutic response to paclitaxel-based chemotherapy in ovarian cancer patients.

To clarify the role of FOXO transcriptional factor in ovarian cancer, screening of FOXO protein expression was performed using western blotting. Among these cells, KFr13Tx, paclitaxel-resistant cell line, only showed marked FOXO1 expression in protein level (Figure 2A). Comparing to FOXO1, FOXO3a and FOXO4 did not show much difference among these cell lines. As speculated, PI3K/Akt activity was considerably lower in KFr13Tx, as reflected by the phosphorylated Akt levels. For further analysis, the transcript levels were also examined by RTQ–PCR, which revealed FOXO1 mRNA level was 15-fold highly expressed in KFr13Tx cells compared to KF28 cells (Figure 2B). These results prompted us to hypothesise that overexpression of FOXO1 in these cell lines correlates especially with the mechanism of paclitaxel resistance.

### Immunohistochemical analysis of FOXO1 expression in ovarian cancer samples from chemotherapy-responded and non-responded patients

To investigate whether our *in vitro* data are relevant to clinical practice, immunohistochemical reactivities of FOXO1 in ovarian cancer samples, obtained at surgery before chemotherapy, with different chemotherapeutic response to paclitaxel-based chemotherapy, were examined. Representative immunohistological staining of responder and non-responder are shown in Figure 2C. FOXO1 overexpression with strong cytoplasmic staining was observed in 5 of 10 non-responders (50%), whereas it was less frequently detected in 2 of 13 responders (15%) (*P*=0.073). Immunoreactivity was not correlated with stage or histological grade (data not shown).

### Induction of FOXO1 by paclitaxel in ovarian cancer cell lines

**Figure 3:**
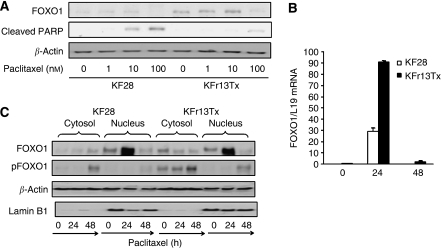
Induction of FOXO1 by paclitaxel in ovarian cancer cell lines. (**A**) KF28 and KFr13Tx cells were incubated with the indicated concentrations of paclitaxel for 24 h and whole cell lysates were probed for FOXO1 and cleaved PARP expression by western blot analysis. *β*-Actin served as a loading control. (**B**) KF28 and KFr13Tx cells treated with 10 nM paclitaxel were harvested at the indicated time points, analysed by RTQ–PCR for FOXO1 mRNA level. The results show mean±s.d. of triplicate measurements. (**C**) KF28 and KFr13Tx cells treated with 10 nM paclitaxel were harvested at the indicated time points and the whole cell lysates were probed for FOXO1 and phospho-FOXO1 expression, and cytosolic and nuclear protein fractions were also probed for FOXO1 expression. *β*-Actin and Lamin B1 served as a loading control.

To investigate further the correlation of FOXO1 and paclitaxel, FOXO1 expression was examined in KF28 and KFr13Tx cells treated with paclitaxel at the increased concentrations for 24 h. Western blotting showed strong induced FOXO1 expression in KFr13Tx cells by paclitaxel treatment, whereas its induction was very weak in KF28 cells (Figure 3A). Conversely, cleaved PARP expression as apoptosis marker was distinctively induced in KF28 cells, whereas its expression was almost undetectable in KFr13Tx cells even at 100 nM concentration, supporting the previous results (Figure 1C and D). Again, RTQ–PCR was performed to examine transcript levels of FOXO1 in both cells treated with 10 nM paclitaxel at the indicated time points. FOXO1 mRNA expression was induced in both cells, which were peaked after 24 h, especially marked in KFr13Tx cells (Figure 3B). For further analysis, translocation of FOXO1 was also investigated using protein fraction by western blotting. Nuclear translocation of FOXO1 was clearly observed in both cells, which were again peaked after 24 h treatment (Figure 3C). The nuclear decrease after 48 h correlates with increase in phosphorylated (Ser256) FOXO1 levels in cytosol. Notably, FOXO1 expression in the cytoplasmic fraction was increased in KFr13Tx cells compared to KF28 cells, whereas nuclear FOXO1 levels were comparable in both cells, which were compatible with the previous results (Figure 2C).

### FOXO1 attenuates sensitivity to paclitaxel-induced cell death in paclitaxel-resistant cell lines

**Figure 4:**
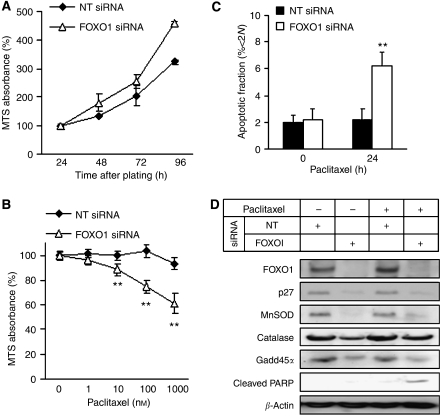
FOXO1 attenuates proliferation and sensitivity to paclitaxel-induced cell death in paclitaxel-resistant cell lines. (**A**) KFr13Tx cells seeded and incubated in 96-well plates for 24 h were transfected with either non-targeting (NT) siRNA or FOXO1 siRNA for 48 h and cell viability was determined by MTS assay at the indicated time points. (**B**) Loss of cell viability was determined by MTS assay in KFr13Tx cells first transfected with non-targeting (NT) siRNA or FOXO1 siRNA and treated later with increasing doses of paclitaxel for 24 h. (**C**) In parallel experiments, KFr13Tx cells transfected with non-targeting (NT) siRNA or FOXO1 siRNA were treated with 10 nM paclitaxel for 24 h and the apoptotic cell fraction was determined by flow cytometry. The results show mean±s.d. of triplicate measurements and ^**^P<0.001. (**D**) Whole cell protein lysates of KFr13Tx cells transfected with non-targeting (NT) siRNA or FOXO1 siRNA and treated with or without 10 nM paclitaxel for 24 h were analysed by western blot for FOXO1, p27^Kip1^, MnSOD, catalase, GADD45*α* and cleaved PARP expression. *β*-Actin served as a loading control.

To clarify the role of FOXO1 in ovarian cancer cells, gene-silencing experiment was performed in KFr13Tx cells. After transfection with either non-targeting siRNA or FOXO1 siRNA, cellular proliferation was monitored by MTS assay at the indicated time points. FOXO1 siRNA slightly promoted cellular proliferation, whose effect was not quite remarkable (Figure 4A). The same silencing experiment was carried out before paclitaxel treatment at the indicated concentrations for 24 h. FOXO1 siRNA considerably increased the sensitivity to paclitaxel as determined by MTS assay (Figure 4B). These findings were again confirmed by FACS analysis using PI staining for 24 h treatment at 10 nM paclitaxel (Figure 4C). FOXO1 silencing followed by paclitaxel treatment in KFr13Tx cells was again performed and putative FOXO target genes involving with cell cycle inhibition (p27^Kip1^), defence against oxidative stress (MnSOD, catalase), and DNA repair (GADD45*α*) were examined by western blotting. Transfection with FOXO1 siRNA decreased expression levels of these target genes, especially in p27^Kip1^ and MnSOD, regardless of paclitaxel treatment (Figure 4D). Notably, cleaved PARP was detectable by paclitaxel treatment only in FOXO1 silencing cells, supporting the previous results (Figure 4B and C).

### Attenuation of oxidative stress by paclitaxel and FOXO1 in ovarian cancer cell lines

**Figure 5:**
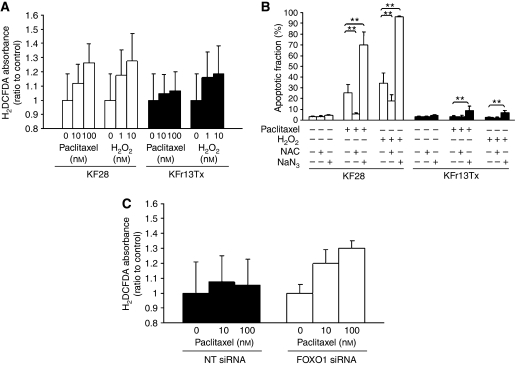
Paclitaxel attenuates oxidative stress in ovarian cancer cell lines. (**A**) After exposure of KF28 and KFr13Tx cells to paclitaxel or H_2_O_2_, a concentration-dependent intracellular increase in H_2_O_2_ levels were detected by spectrofluorometry. (**B**) Concomitant treatment with *N*-acetylcysteine (NAC), H_2_O_2_ scavenger, or NaN_3_, catalase inhibitor, decreased or increased paclitaxel or H_2_O_2_ induced cell death. The results show mean±s.d. of triplicate measurements and ^**^P<0.001. (**C**) KFr13Tx cells transfected with non-targeting (NT) siRNA or FOXO1 siRNA were exposed to paclitaxel and intracellular increase in H_2_O_2_ levels were detected by spectrofluorometry.

To investigate the possible underlying mechanism that FOXO1 attenuates paclitaxel sensitivity in ovarian cancer cells, intracellular reactive oxygen species (ROS) induced by paclitaxel was measured in KF28 cells and KFr13Tx cells. As assessed by C-H_2_DCFDA fluorescence, intracellular H_2_O_2_ levels were increased in KF28 cells when exposed for 4 h to increasing concentrations of paclitaxel or H_2_O_2_ as indicated, whereas those changes were not marked in KFr13Tx cells exposed with paclitaxel (Figure 5A). To study further the role of ROS accumulation in paclitaxel cytotoxicity, the effects of co-incubation of 10 nM paclitaxel or 500 *μ*M H_2_O_2_ for 24 h with antioxidant, 400 *μ*M N-acetylcysteine (NAC), H_2_O_2_ scavenger, or 1 mM NaN_3_, inhibitor of catalase, were investigated in both cells by Trypan blue exclusion test. Co-treatment with NAC or NaN_3_ in KF28 cells significantly decreased or increased paclitaxel or H_2_O_2_ induced cell death, whereas co-treatment with NaN_3_ in KFr13Tx cells also increased paclitaxel or H_2_O_2_ induced cell death (Figure 5B). On the basis of these results, the possibility whether FOXO1 attenuates paclitaxel-induced cytotoxicity through oxidative stress was studied again by ROS measurement in KFr13Tx cells using silencing experiment. As shown in Figure 5C, intracellular H_2_O_2_ levels were increased in KFr13Tx cells transfected with FOXO1 siRNA compared to those with NTsiRNA when exposed for 4 h to increasing concentrations of paclitaxel as indicated.

### MnSOD expression in paclitaxel-sensitive and -resistant ovarian cancer cell lines and ovarian cancer samples

**Figure 6:**
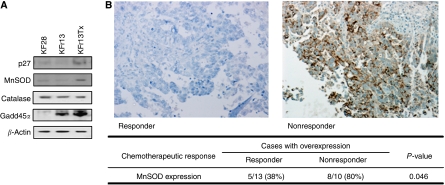
Differential expression of MnSOD in drug-sensitive and -resistant ovarian cancer cell lines and ovarian cancer samples. (**A**) Comparative analysis of p27^Kip1^, MnSOD, catalase, GADD45*α* expression in KF28, KFr13 and KFr13Tx cells by western blot analysis. *β*-Actin served as a loading control. (**B**) The representative immunohistological staining of MnSOD of a chemotherapy-responder and non-responder is shown. The adjacent table summarises the number of patients with immunohistochemical reactivity of MnSOD according to the chemotherapeutic response to paclitaxel-based chemotherapy in ovarian cancer patients.

To determine further the relevance of FOXO1 target genes in ovarian cancer cells, we also compared the levels of p27^Kip1^, MnSOD, catalase and GADD45*α* expression in KF28, KFr13 and KFr13Tx cells by western blotting. As shown in Figure 6A, p27^Kip1^ and MnSOD were strongly expressed especially in paclitaxel-resistant cell line, whereas GADD45*α* expression was also comparably observed in KFr13 cells and catalase expressions were almost the same among these three cell lines. Together with the previous results, we speculated that the FOXO1 attenuates paclitaxel sensitivity through control of oxidative stress by regulation of MnSOD. Finally, again to investigate whether our *in vitro* data is relevant to clinical practice, immunohistochemical reactivities of MnSOD in the same ovarian cancer samples were examined. Representative immunohistological staining of responder and non-responder are shown in Figure 6B. MnSOD overexpression with strong cytoplasmic staining was observed in 8 of 10 non-responders (80%), whereas it was less frequently detected in 5 of 13 responders (38%) (*P*=0.046). Furthermore, the cases with overexpression of FOXO1 also showed MnSOD overexpression in non-responder patients.

## DISCUSSION

Although most ovarian cancers are responsive to paclitaxel-based chemotherapy, the emergence of drug-resistant cancer clones can lead to treatment failure and disease relapse. There have been several reports regarding overexpression of genes related to paclitaxel resistance. MDR-1 overexpression in ovarian cancer cell lines with paclitaxel resistance had been reported (Masanek *et al*, 1997; Duan *et al*, 1999). Similarly, we had also confirmed MDR-1 overexpression in paclitaxel-resistant derivative ovarian cancer cell line, KFr13_TX_ cells (Yamamoto *et al*, 2000b; Goto *et al*, 2006). The molecular mechanism of MDR-1 is still uncertain. Some studies showed MDR-1 as a predictive marker of poor chemotherapeutic response (Yokoyama *et al*, 1999; Penson *et al*, 2004), but others did not show (Baird and Kaye, 2003; Vasey, 2003; Mozzetti *et al*, 2005).

In the present study, paclitaxel-resistant derivative cells showed increased expression of FOXO1, compared to parent cells and cisplatin-resistant derivative cells. Notably, cytoplasmic FOXO1, which is likely to be inactive and should have no affect on expression of target genes in stress response, was strongly expressed in resistant cells both in cancer cell lines and clinical samples. In contrast, induction and nuclear FOXO1 was markedly induced by 24 h exposure of paclitaxel in both sensitive and resistant cells. It is possible that acute exposure to paclitaxel leads to FOXO1-dependent activation of a proapoptotic gene programme, and that prolonged or chronic exposure promotes selection of cells with another transcriptionally activated gene settings by FOXO1, which are involved in cellular survival and drug resistance. For instance, despite of the several reports showing reduction in Akt phosphorylation in response to paclitaxel, phosphorylation and nuclear exclusion of FOXO1 was clearly observed after 48 h in our experiments, especially stronger in resistant cells. Since FOXO1 has recently been shown to enhance Akt phosphorylation in hepatocytes by repressing the expression of tribble 3 (Trb3), a pseudokinase capable of binding Akt and inhibiting its phosphorylation (Matsumoto *et al*, 2006), it seems to be interesting to investigate whether feedback to PI3K/AKT pathway by FOXO1 could contribute to the survival advantage and development of drug resistance. Although drug resistance in cancer should be multifactorial, it is well recognised that a slower growth rate represents one component of drug resistance. In our results, FOXO1 silencing decreased expression level of p27^Kip1^, which is one FOXO1 target gene involving cell cycle inhibition (Dijkers *et al*, 2000). However, cellular proliferation was not actually attenuated in these cells, which suggests more critical event other than cell growth retardation is involved in these settings.

ROS are thought to play multiple roles including tumour initiation, progression and maintenance, and ROS production is highly increased in cancer cells (Szatrowski and Nathan, 1991; Burdon, 1995). ROS levels fluctuate in response to intracellular as well as extracellular signals and, in turn, stimulate specific signalling cascades, such as MAPKs, that regulate cell growth and cell death (Benhar *et al*, 2001; Davis *et al*, 2001; Tobiume *et al*, 2001). ROS levels are increased in cells exposed to various stress agents, including paclitaxel and other anticancer drugs (Varbiro *et al*, 2001; Ramanathan *et al*, 2005). Agents that decrease ROS can suppress taxol-induced cytotoxicity, whereas increase of ROS levels by inhibition of SOD or glutamylcysteine synthase can enhance taxol-induced cytotoxicity in cancer cell lines (Ramanathan *et al*, 2005). The cellular responses to paclitaxel involve activation of MAPK pathways (Bacus *et al*, 2001). Higher ROS levels and SAPK (stress-activated protein kinases) JNK activity were measured in tumour cells that were sensitive to anticancer agents than in those that were drug-resistant, suggesting that ROS-mediated JNK and p38 activation played a key role in the sensitisation to stress signals and to anticancer drugs (Benhar *et al*, 2001; Davis *et al*, 2001). Thus, control of endogenous ROS level and the regulation of MAPK pathway may involve in proliferation and sensitivity to stress stimuli including anticancer drugs in cancer cells. In the present study, the increase of intracellular H_2_O_2_ levels in ovarian cancer cells were observed by adding extracellular H_2_O_2_ as well as paclitaxel. In addition, modifying intracellular ROS level by co-incubation with NAC or catalase inhibitor showed significant decrease or increase in cytotoxicity of H_2_O_2_ as well as paclitaxel. Moreover, FOXO1 silencing attenuated intracellular H_2_O_2_ levels, and also decreased expression of its putative target gene, MnSOD and Gadd45*α*, simultaneously showing increased paclitaxel-induced cytotoxicity, which collectively suggest one of possible explanation in transcriptional role of FOXO1 as redox mechanism to cytotoxic stimuli such as paclitaxel in these cells.

We also demonstrated in clinical samples that FOXO1 overexpression was correlated with paclitaxel resistance, although the number of samples was small and further analysis will be required to confirm these findings. Among the FOXO1 target genes we examined, MnSOD was strongly expressed especially in paclitaxel-resistant cell line, which prompted us to speculate that FOXO1 might attenuate paclitaxel sensitivity through control of oxidative stress by regulation of MnSOD, then confirmed its overexpression in the same samples showing FOXO1 overexpression from chemoresistant patients. There are far more mechanisms to elucidate although, together with our *in vitro* data, FOXO1 might be the candidate to predict the chemotherapeutic response and it could be a molecular target for the treatment of drug-resistant ovarian cancers.
